# Integrated Enzyme‐ and Ultrasound‐Assisted Extraction of Fennel Seed Proteins: Process Optimization, Structure, and Functionality

**DOI:** 10.1002/fsn3.72010

**Published:** 2026-06-14

**Authors:** Izzet Turker, Hilal Isleroglu

**Affiliations:** ^1^ Faculty of Engineering and Architecture, Food Engineering Department Tokat Gaziosmanpasa University Tokat Turkey

**Keywords:** enzymatic extraction, fennel seed proteins, functional properties, structural properties, ultrasound

## Abstract

In this study, proteins were extracted from fennel seeds ( *Foeniculum vulgare* ) using an integrated enzyme‐assisted and ultrasound‐assisted extraction approach. Enzymatic pretreatment was optimized to maximize protein extraction yield while promoting cell wall degradation, as reflected by reducing sugar release. Following optimization, ultrasound‐assisted extraction was applied as an alternative to classical alkaline extraction to shorten processing time and enhance extraction efficiency. The optimized enzymatic pretreatment conditions were determined as pH 4.20, 48.95°C, enzyme dosage of 1.91 FBGU/g seed, and a treatment time of 2.60 h. Under these conditions, protein extraction yield increased from ~61% in the control sample to ~92% after enzymatic pretreatment followed by classical alkaline extraction, which required 1 h of processing. Notably, within only 10 min of ultrasound‐assisted extraction following enzymatic pretreatment, a slightly higher yield (~93%) was achieved. Functional characterization revealed that enzyme–ultrasound‐treated protein isolates exhibited improved solubility, emulsifying, foaming, and water/oil‐holding properties compared to control samples. Molecular weight profiling (SDS‐PAGE), structural features assessed by FT‐IR spectroscopy, and thermal properties evaluated by differential scanning calorimetry collectively indicated that the applied treatments did not cause noticeable degradation of protein subunits, while subtle conformational modifications were associated with improved functional performance and enhanced thermal stability, as reflected by the increase in denaturation temperature from 143.28°C to 176.23°C. Amino acid profiling demonstrated a balanced essential amino acid composition, highlighting the nutritional quality of fennel seed protein isolate. The results demonstrate that the combination of enzymatic pretreatment and ultrasound‐assisted extraction is an effective and sustainable strategy for producing functional fennel seed protein isolates suitable for food applications.

## Graphical abstract

This graphical abstract presents an integrated enzyme‐ (Viscozyme L) and ultrasound‐assisted extraction strategy that successfully boosts fennel seed protein yield from 61% to 93%. Following process optimization, the resulting protein isolate exhibits significantly enhanced solubility, emulsifying, foaming, and water/oil‐holding properties. Advanced characterizations via SDS‐PAGE, FT‐IR, and DSC confirm that this integrated process improves thermal stability and induces moderate structural rearrangements while preserving a nutritionally valuable amino acid profile, making it a promising functional plant protein ingredient.

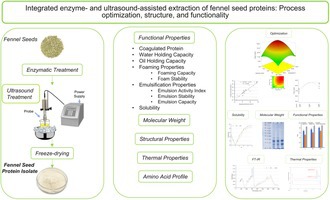

## 1 Introduction

Plant‐derived proteins have attracted increasing interest as functional and sustainable food ingredients, driven by environmental constraints of animal agriculture and the growing demand for alternative protein sources (Munialo 2024; Navarré et al. 2025). In addition to their nutritional contribution, plant proteins play a key technological role in food systems through properties such as solubility, emulsification, foaming behavior, and water and oil retention, which collectively determine texture, stability, and consumer acceptance (Tang et al. 2024; Yeasmen and Orsat 2025).

Despite these advantages, the nutritional quality of plant proteins is often limited by their essential amino acid composition and digestibility. In many plant sources, lysine, methionine, and tryptophan are present at suboptimal levels, reducing overall protein quality when compared to animal‐derived proteins (Berrazaga et al. 2019). Protein quality assessment tools such as PDCAAS and DIAAS highlight this gap, with most plant proteins exhibiting lower scores than egg or milk proteins, although soy and canola represent notable exceptions (Balandrán‐Quintana et al. 2019; FAO 2013). Improving extraction efficiency and preserving amino acid integrity are therefore essential steps toward enhancing the nutritional and functional value of plant protein isolates.

From an environmental perspective, plant proteins offer substantial benefits over animal proteins. The production of animal‐derived protein requires significantly higher land, water, and energy inputs, while contributing disproportionately to greenhouse gas emissions (Sabaté and Soret 2014). Consequently, the valorization of plant materials that are currently underexploited as protein sources has become an important research focus. In this context, fennel ( *Foeniculum vulgare* ), a crop traditionally valued for its aromatic seeds and medicinal properties, represents a promising candidate. Fennel seeds are also known for their rich biochemical composition, including essential oils, phenolic acids, flavonoids, proteins, and other bioactive compounds. Their antioxidant potential has been demonstrated using different in vitro assays, including radical scavenging, reducing power, metal chelating, and peroxide scavenging tests (Barakat et al. 2022; Oktay et al. 2003). The bioactive composition of fennel may vary depending on genotype, plant type, geographical origin, and cultivation conditions. In this regard, Faudale et al. (2008) reported that wild fennel exhibited higher radical scavenging activity as well as higher total phenolic and flavonoid contents than medicinal and edible fennel samples, while Khammassi et al. (2022) showed considerable variation in phenolic profiles and antioxidant potential among wild fennel populations. Although fennel seeds contain appreciable protein levels (≈17%–18%) (Ehsanipour et al. 2012), research has largely concentrated on their essential oil and phenolic fractions, leaving their protein potential insufficiently explored. To date, only limited studies have addressed fennel seed proteins, primarily focusing on crude protein fractions without systematic evaluation of structural or functional attributes (Mohana Dass et al. 2019).

Protein extraction methodology strongly influences yield, molecular structure, and downstream functionality (Kumar et al. 2021). Conventional alkaline extraction remains widely used due to its simplicity and high efficiency, yet harsh pH conditions may induce chemical modifications and impair protein quality (del Mar Contreras et al. 2019). To overcome these drawbacks, enzyme‐assisted extraction (EAE) has been proposed as a mild and selective approach, enabling protein release through targeted degradation of cell wall polysaccharides (Perović et al. 2020; Rashwan et al. 2025). In parallel, ultrasound‐assisted extraction (UAE) has emerged as an effective physical technique that enhances mass transfer via cavitation, thereby accelerating extraction kinetics and improving functional performance (Dzah et al. 2020).

Recent studies indicate that combining enzymatic pretreatment with UAE can generate synergistic effects, resulting in higher protein recovery, reduced processing time, and improved techno‐functional properties (Görgüç et al. 2020; Yang et al. 2024; Yeasmen and Orsat 2024). Although such integrated strategies have been successfully applied to several plant matrices, their application to fennel seeds has not yet been systematically investigated, particularly with respect to protein structure, thermal behavior, and nutritional quality.

Therefore, the objective of this study was to optimize protein extraction from fennel seeds using an integrated enzyme‐assisted and ultrasound‐assisted approach. EAE conditions were optimized using response surface methodology, and the resulting protein isolates were comprehensively characterized in terms of technofunctional properties, including solubility, hydration behavior, emulsifying, and foaming properties. In addition, amino acid composition and amino acid scores were determined to evaluate nutritional quality, while molecular weight distribution (sodium dodecyl sulfate–polyacrylamide gel electrophoresis [SDS‐PAGE]), secondary structure (Fourier transform infrared [FT‐IR]), and thermal behavior (differential scanning calorimetry [DSC]) were analyzed to elucidate structure–function relationships. This study provides the first detailed characterization of fennel seed protein isolates and highlights their potential as sustainable and functional ingredients for food applications.

## 2 Materials and Methods

### 2.1 Material

Food‐grade fennel ( *F. vulgare* ) seeds were obtained from local producers in Tokat, Türkiye. Foreign materials were removed manually, and the seeds were milled using a laboratory scale blender (Sinbo SCM 934, Türkiye) and sieved through a 630 μm mesh. The ground seeds were defatted with n‐hexane at a sample‐to‐solvent ratio of 1:4 (w/v) for 30 min at room temperature, and the extraction was repeated three times with fresh solvent, following a modified procedure based on Feyzi et al. (2015). Defatted samples were stored at −18°C in sealed containers until use. The protein content of the defatted fennel seed flour, determined by the Kjeldahl method using a nitrogen‐to‐protein conversion factor of 6.25, was 20.57% ± 0.38% (w/w), and the same batch was used throughout the study.

### 2.2 Enzyme‐Assisted Pretreatment and Experimental Design

A 29‐run, four‐factor Box–Behnken design was employed to investigate the enzymatic pretreatment parameters affecting protein extraction from fennel seeds. Based on preliminary experiments, the design was constructed with the following independent variable ranges: pH (3.0–5.0), temperature (35°C–55°C), enzyme dosage (0.25–2.50 FBGU/g seed), and incubation time (1‐3 h). The pH of the fennel seed dispersions was adjusted to the target values using 0.1 M HCl or 0.1 M NaOH and monitored during the treatment, with readjustment when necessary. Viscozyme L (Novozymes, Sigma‐Aldrich, Germany), a multi‐enzyme formulation containing cellulase, hemicellulase, and pectinase, was selected due to its ability to degrade key components of the fennel seed cell wall matrix. This enzymatic action facilitates the breakdown of cellulose, hemicellulose, and pectin, allowing the release of intracellular proteins and thereby improving extraction efficiency.

After enzymatic pretreatment, the pH of the mixtures was adjusted to 10.0 using 1 M NaOH and monitored with a pH meter (WTW Inolab pH 7110, Germany), and the samples were stirred for 1 h. The alkaline‐treated mixtures were then centrifuged at 6000 rpm (in a 1620 A rotor, Hettich Universal 320 R, Andreas Hettich GmbH & Co. KG, Tuttlingen, Germany) for 15 min, and the resulting supernatants were collected. Protein content in the supernatants was determined by the Kjeldahl method.

Protein yield was computed by comparing the amount of protein obtained in the extract with the total protein initially present in the ground and defatted fennel seeds, as shown in Equation (1):

(1) $$\text{Protein yield}\;\left(\%\right)=\frac{\text{Protein quantity of the extract}\;\left(g\right)}{\;\text{Protein quantity of the ground and defatted fennel seeds}\;\left(g\right)}\times 100$$

In addition, reducing sugars in the extracts were quantified using the dinitrosalicylic acid (DNS) method and expressed as mg glucose per g seed. Both protein extraction yield and reducing sugar content were evaluated as response variables, and optimal conditions were identified using the desirability function approach. The experimental results and responses are summarized in Table 1.

**TABLE 1:** Experimental design and results.

Run	pH	Temperature (°C)	Enzyme dosage (FBGU/g seed)	Time (h)	Protein yield (%)	Reducing sugar (mg glucose/g seed)
1	3	35	1.375	2	72.67 (±1.11)	43.23 (±0.39)
2	5	45	1.375	3	84.45 (±1.95)	78.51 (±0.44)
3	5	35	1.375	2	66.04 (±1.77)	52.94 (±0.82)
4	4	45	0.25	1	69.68 (±0.10)	34.44 (±0.45)
5	3	55	1.375	2	70.78 (±2.03)	45.62 (±0.69)
6	5	45	1.375	1	71.50 (±1.22)	57.31 (±0.26)
7	3	45	1.375	1	63.25 (±1.11)	35.74 (±0.19)
8	3	45	2.5	2	70.35 (±0.64)	55.97 (±0.19)
9	4	45	2.5	1	79.87 (±0.94)	52.39 (±0.26)
10	4	45	1.375	2	88.67 (±0.75)	91.12 (±0.39)
11	4	55	1.375	1	77.88 (±0.71)	62.53 (±0.32)
12	5	45	2.5	2	71.51 (±1.51)	79.01 (±0.44)
13	4	45	2.5	3	91.16 (±0.66)	96.51 (±0.25)
14	4	45	0.25	3	72.19 (±0.60)	33.64 (±0.53)
15	4	45	1.375	2	88.19 (±1.07)	90.32 (±0.45)
16	4	55	1.375	3	90.57 (±1.80)	88.89 (±0.19)
17	4	35	0.25	2	69.85 (±0.25)	26.03 (±0.44)
18	5	45	0.25	2	71.00 (±1.22)	33.60 (±0.51)
19	4	35	1.375	3	73.80 (±0.81)	67.36 (±0.13)
20	3	45	1.375	3	71.91 (±0.05)	47.85 (±1.05)
21	4	45	1.375	2	89.10 (±0.15)	93.10 (±0.79)
22	4	35	2.5	2	74.45 (±0.07)	74.72 (±0.32)
23	4	55	2.5	2	78.47 (±0.22)	81.33 (±0.19)
24	4	45	1.375	2	85.10 (±0.87)	92.34 (±0.70)
25	3	45	0.25	2	62.34 (±1.06)	15.43 (±0.39)
26	5	55	1.375	2	88.15 (±0.08)	86.58 (±0.44)
27	4	45	1.375	2	90.96 (±0.56)	89.90 (±0.44)
28	4	35	1.375	1	72.58 (±1.05)	62.28 (±0.26)
29	4	55	0.25	2	74.47 (±1.23)	50.92 (±0.26)

### 2.3 Production of Protein Isolates

Three distinct types of protein isolates were obtained in this study. The first isolate served as the control (C) and was produced through a conventional alkaline extraction process, without any enzymatic pretreatment. The second isolate was derived by subjecting the fennel seed samples to enzymatic pretreatment under the optimized conditions, followed by classical alkaline extraction (EA). The third isolate involved a combined treatment, where enzymatic pretreatment was followed by ultrasound‐assisted extraction (EAU), replacing the classical extraction step. Sonication was performed using a probe‐type ultrasonic processor (QSonica Q500, 500 W, 20 kHz, USA) equipped with a ½″ (12.7 mm) titanium probe. The treatment was conducted at 50% amplitude for 10 min under pulsed conditions (4 s on/1 s off; *α* = 0.8). To limit excessive heating, the sample beaker was placed in an ice‐water bath, and the extraction temperature was maintained at approximately 25°C throughout sonication. The total energy delivered during sonication was approximately 24 kJ, corresponding to an effective power intensity of ~34.5 W/cm^2^ after correction for background power.

Following all extraction procedures, the resulting supernatants were adjusted to pH 4.0 using 0.1 M and 1 M HCl to induce isoelectric precipitation of fennel seed proteins. The precipitation process was carried out at room temperature and allowed to proceed for 6 h. The precipitated proteins were then collected by centrifugation at 9000 rpm for 60 min at 25°C. The supernatants were discarded, and the precipitates were washed three times with acidified distilled water. The washed protein fractions were redissolved in distilled water adjusted to pH 7.2 and subsequently freeze‐dried using a laboratory freeze dryer (Christ Alpha 1–4 LSC Plus, Germany) at −18°C for 72 h. The resulting protein powders were stored in sealed containers at −18°C until further analyses.

### 2.4 Functional Properties of Protein Isolates

To comprehensively evaluate the functional properties of the protein isolates obtained through different extraction strategies, a series of physicochemical and structural analyses were conducted. These included the determination of coagulated protein content, water and oil holding capacities, foaming properties (foam capacity [FC] and foam stability [FS]), and emulsification parameters (emulsion activity index [EAI], emulsion stability [ES], and emulsion capacity [EC]). Additionally, solubility behavior across different pH values was assessed. Unless otherwise stated, freeze‐dried protein isolates were reconstituted in distilled water or the buffer specified for each analysis. The pH, buffer composition, and protein concentration used for each assay are provided in the corresponding method sections. Functional property measurements were performed using defined amounts of freeze‐dried protein isolate powder according to the corresponding methods, while the soluble protein fraction was directly quantified in the solubility assay.

#### 2.4.1 Coagulated Protein

The percentage of coagulated protein was determined according to the method of Kramer and Kwee (1977) with minor modifications. Briefly, 0.2 g of protein isolate was dispersed in 10 mL of 0.025 M citrate–phosphate buffer (pH 7.0) and centrifuged at 6000 rpm for 10 min. Aliquots of the supernatant were heated at 100°C for 15 min, and protein contents before (*A*_1_) and after (*A*_2_) heat treatment were measured spectrophotometrically at 540 nm using the Biuret method (Shimadzu UV‐1800, Japan). The coagulated protein percentage was calculated as follows:

(2) $$\text{Coagulated protein}\;\left(\%\right)=\frac{A_{1}-A_{2}}{A_{1}}\times 100$$

#### 2.4.2 Water and Oil Holding Capacities

Water‐holding capacity (WHC) and oil‐holding capacity (OHC) of the protein isolates were determined using a modified method of Vinayashree and Vasu (2021). Briefly, 250 mg of protein isolate was mixed with 15 mL of distilled water or olive oil, vortexed for 1 min, and allowed to stand at room temperature for 1 h. The samples were then centrifuged at 3000 rpm for 20 min, and the supernatant was discarded. WHC and OHC were expressed as grams of water or oil retained per gram of dry protein isolate (g/g).

#### 2.4.3 Foaming Properties

FC and FS were evaluated according to Timilsena et al. (2016). Protein dispersions (20 g/L) were homogenized using an Ultra‐Turrax homogenizer (IKA T‐18, Germany) at 10,000 rpm for 5 min. The initial volume (*V*_0_), volume immediately after foaming (*V*_1_), and volume after 1 h (*V*_2_) were recorded. FC and FS were calculated as:

(3) $$\text{Foaming capacity}\;\left(\%\right)=\frac{V_{1}-V_{0}}{V_{0}}\times 100$$

(4) $$\text{Foam stability}\;\left(\%\right)=\frac{V_{2}-V_{0}}{V_{1}-V_{0}}\times 100$$

#### 2.4.4 Emulsification Properties

EAI, ES, and EC were determined using a modified turbidity‐based method (Feyzi et al. 2017). Briefly, 22.5 mg protein isolate was dispersed in 4.5 mL phosphate buffer (pH 7.0), vortexed for 1 min, mixed with 1.5 mL sunflower oil, and homogenized at 22,000 rpm for 2 min. For ES, 250 μL of freshly prepared emulsion (*t* = 0) was diluted in 50 mL of 1 g/L SDS solution and absorbance at 500 nm was recorded immediately (*A*_0_) and after 15 min (*A*_15_); ES was calculated as:

(5) $$\text{Emulsion stability}\;\left(\min\right)=\frac{A_{0}}{\left(A_{0}-A_{15}\right)}\times t$$

where *t* = 15 min.

For EAI, turbidity was calculated as $T=2.303\;A_{0}/L$, and EAI was expressed as interfacial area per unit protein:

(6) $$\text{Emulsion activity index}\;\left(m^{2}/g\right)=\frac{2T\times D}{\Phi \times C}=\frac{\left(2\times 2.303\times A_{0}\times D\right)}{\left(\Phi \times C\times L\right)}$$

where *D* is the dilution factor (200), Φ is the volumetric oil fraction, *C* is the protein concentration (0.005 g/mL), and *L* is the cuvette path length (10^−2^ m). EC was determined by homogenizing equal volumes of 1.0% (w/v) protein solution and sunflower oil at 7200 rpm for 2 min, centrifuging at 3250 rpm for 2 min, and calculating:

(7) $$\text{Emulsion capacity}\;\left(\%\right)=\frac{H_{1}}{H_{0}}\times 100$$

#### 2.4.5 Solution Properties

The solubility of the protein isolates (g/L) was determined using a modified method described by Huang et al. (2020). Protein isolate dispersions were prepared in distilled water at a concentration of 15 g/L, and the pH was adjusted to values ranging from 2.0 to 12.0 using either HCl or NaOH. The samples were stirred for 30 min at room temperature and subsequently centrifuged at 6000 rpm for 15 min. The protein content of the supernatant was then used to calculate solubility.

### 2.5 Molecular, Structural, and Thermal Properties of Protein Isolates

The molecular weight distribution of the protein isolates was evaluated to identify possible aggregation or degradation effects induced by the applied treatments. Structural features were further examined using FT‐IR spectroscopy to assess changes in secondary structure. In addition, thermal properties, including denaturation temperature and denaturation enthalpy, were determined by DSC to evaluate the thermal stability of the protein isolates.

#### 2.5.1 Molecular Weight Distribution (SDS‐PAGE)

The molecular weight distribution of the protein isolates was determined by SDS‐PAGE according to the method of Laemmli (1970). Electrophoretic separation was performed using a vertical gel electrophoresis system (Hoefer SE 260, USA), and proteins were visualized by Coomassie Brilliant Blue R‐250 staining. A 12% resolving gel was used, and protein samples were loaded at 50 μg per lane. Electrophoresis was conducted at a constant voltage of 200 V for 50 min. A pre‐stained molecular weight marker (Thermo Fisher PageRuler, 10–180 kDa) was used as the reference standard.

#### 2.5.2 Structural Properties

The structural properties of the protein isolates were analyzed by FT‐IR spectroscopy using a PerkinElmer Spectrum 400 instrument (USA), following the approach described by Byler and Susi (1986). Spectra were recorded using the diamond attenuated total reflectance (ATR) accessory over the range of 4000–400 cm^−1^. The secondary structure composition of the protein isolates was evaluated based on the Amide I region (1600–1700 cm^−1^) of the FT‐IR spectra. Deconvolution and peak fitting of the Amide I band were performed using PeakFit software (v4.12, Systat Software, USA).

#### 2.5.3 Thermal Properties

The thermal properties of protein isolates were determined using DSC (PerkinElmer DSC 8000, USA). Denaturation temperature (*T*_d_, °C) and denaturation enthalpy (Δ*H*_d_, J/g) were evaluated under a nitrogen atmosphere. Ten milligrams of each sample was heated from 20°C to 200°C at a constant heating rate of 5°C/min.

### 2.6 Amino Acid Composition

The amino acid composition of the protein isolate obtained using enzymatic pretreatment followed by UAE was analyzed to determine its nutritional potential (EAU sample). Acid hydrolysis was applied prior to analysis, and amino acid profiles were determined using ultra‐fast liquid chromatography with ultraviolet detection (UFLC–UV) (Shimadzu 20A, Japan). The contents of aspartic acid, glutamic acid, serine, glycine, histidine, arginine, threonine, alanine, proline, tyrosine, valine, methionine, isoleucine, leucine, phenylalanine, lysine, and tryptophan were quantified. Tryptophan content was separately determined using high‐performance liquid chromatography with fluorescence detection (HPLC–FLD) (Shimadzu 20A, Japan). Amino acid scores were calculated by comparing the essential amino acid contents of the fennel seed protein isolate with those of the reference protein.

### 2.7 Statistical Analysis

Regression analysis, statistical analyses, contour plots, response surface graphs, and optimization processes to determine the effects of all process variables were carried out using the Design Expert 7.0 (Stat‐Ease Inc., USA) software. The model used for regression analysis is provided in Equation (8).

(8) $$\text{Extraction yield (\%)}\;or\;\;\text{Reducing sugar}\;\left(\mathrm{mg}\;\text{glucose}/g\;\text{seed}\right)=\;\beta_{0}+\sum_{i=1}^{k}\beta_{i}X_{i}+\sum_{i=1}^{k}\beta_{ii}X_{i}^{2}+\sum_{i=1}^{k-1}\sum_{j=i+1}^{k}\beta_{\mathrm{ij}}X_{i}X_{j},k=1,2,3,4$$

where *β*_0_, *β*_*i*_, *β*_*ii*_, and *β*_*ij*_ are the coefficients, *X* is the independent variable, and *k* is the number of independent variables.

## 3 Results and Discussion

### 3.1 Optimization of Enzymatic Pretreatment

The experimental design matrix and the corresponding responses obtained for enzyme‐assisted protein extraction from fennel seeds are presented in Table 1. Protein extraction yield and reducing sugar content varied markedly depending on the pretreatment conditions, and a clear positive association between these two responses was observed across the experimental domain. The highest protein extraction yield (91.16% ± 0.66%) and reducing sugar content (96.51 ± 0.25 mg glucose/g seed) were obtained at pH 4.0, 45°C, an enzyme dosage of 2.5 FBGU/g seed, and a treatment time of 4 h. In contrast, the lowest values for both protein yield (62.34% ± 1.06%) and reducing sugar content (15.43 ± 0.39 mg glucose/g seed) were recorded under acidic conditions (pH 3.0) combined with the lowest enzyme dosage (0.25 FBGU/g seed) and a shorter treatment time (3 h). These results indicate that insufficient enzymatic activity limits both carbohydrate degradation and protein release from the fennel seed matrix.

At constant enzyme dosage and treatment time, increasing the extraction temperature led to higher protein yields and reducing sugar contents at all pH levels. This behavior can be attributed to enhanced mass transfer and increased solubility of cell wall components at elevated temperatures, which facilitates enzyme accessibility to polysaccharide substrates. Similar temperature‐dependent improvements in enzyme‐assisted protein extraction have been reported for oilseed and cereal by‐products (Guan and Yao 2008; Zhang et al. 2009). Likewise, increasing enzyme dosage under fixed temperature and time conditions resulted in a pronounced increase in both responses, particularly at pH 4.0, highlighting the critical role of carbohydrase concentration in disrupting the fennel seed cell wall structure.

The adequacy of the developed quadratic models for protein extraction yield and reducing sugar content was confirmed by ANOVA (Table 2). The models were statistically significant (*p* < 0.05), while the lack‐of‐fit values were not significant (*p* > 0.05), indicating a good agreement between experimental and predicted values. All linear terms (pH, temperature, enzyme dosage, and treatment time) significantly affected both responses (*p* < 0.05). Moreover, quadratic effects of all variables were significant for protein extraction yield, whereas the quadratic terms of pH, enzyme dosage, and treatment time significantly influenced reducing sugar content. Selected interaction terms also contributed significantly, demonstrating the complex interdependence of process variables during enzymatic pretreatment.

**TABLE 2:** ANOVA table.

Source	SD	Sum of squares		*F*		*p*	
		Protein yield	Reducing sugar	Protein yield	Reducing sugar	Protein yield	Reducing sugar
Model	14	1980.53	15540.61	10.89	150.59	< 0.0001	< 0.0001
*X*_1_	1	142.46	1730.56	10.96	234.77	0.0051	< 0.0001
*X*_2_	1	216.21	664.76	16.64	90.18	0.0011	< 0.0001
*X*_3_	1	178.47	5037.61	13.73	683.40	0.0024	< 0.0001
*X*_4_	1	202.64	973.25	15.59	132.03	0.0015	< 0.0001
*X*_1_ *X*_2_	1	144.09	244.04	11.09	33.11	0.0050	< 0.0001
*X*_1_ *X*_3_	1	14.07	5.95	1.08	0.81	0.3157	0.3842
*X*_1_ *X*_4_	1	4.60	20.62	0.35	2.80	0.5613	0.1166
*X*_2_ *X*_3_	1	0.091	83.65	6.99 × 10^−3^	11.35	0.9346	0.0046
*X*_2_ *X*_4_	1	32.90	113.18	2.53	15.35	0.1339	0.0015
*X*_3_ *X*_4_	1	19.32	504.22	1.49	68.40	0.2429	< 0.0001
*X*_1_^2^	1	748.90	3578.81	57.63	485.50	< 0.0001	< 0.0001
*X*_2_^2^	1	164.01	605.25	12.62	82.11	0.0032	< 0.0001
*X*_3_^2^	1	422.91	3496.00	32.54	474.27	< 0.0001	< 0.0001
*X*_4_^2^	1	97.87	1059.74	7.53	143.76	0.0158	< 0.0001
Residual	14	181.94	103.20				
Lack of fit	10	163.89	95.96	3.63	5.30	0.1127	0.0611
Pure error	4	18.05	7.24				
Total	28	2162.47	15643.81				

Abbreviation: DF: degree of freedom, *X*_1_: pH, *X*_2_: temperature (°C), *X*_3_: enzyme dosage (FBGU/g seed), *X*_4_: time (h).

The adequacy of the developed models was evaluated using statistical indicators. For protein extraction yield, the model showed good agreement between experimental and predicted values, with an *R* ^2^ of 0.9159 and an adjusted *R* ^2^ of 0.8317. The adequate precision value (11.49), which exceeded the recommended minimum of 4, together with a low coefficient of variation (CV = 4.69%), indicated satisfactory model precision and reliability. Similarly, the reducing sugar model exhibited excellent predictive performance, as reflected by high *R* ^2^ (0.9934) and adjusted *R* ^2^ (0.9868) values, a very high adequate precision (42.36), and a low CV value (4.33%), confirming the robustness of the model.

**FIGURE 1:**
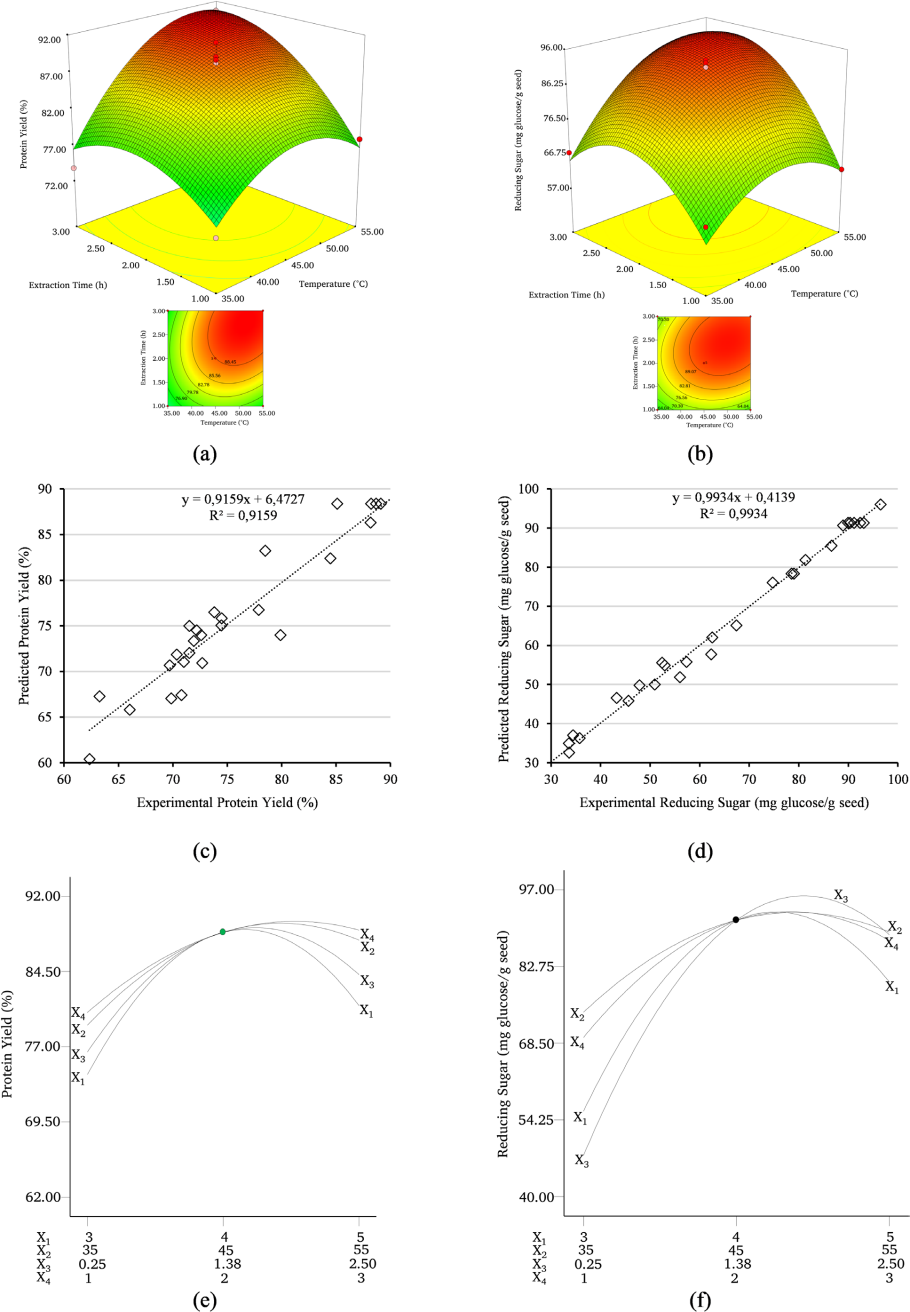
3D response surface graphs at pH 4.0 and 1.38 FBGU/g seed enzyme dosage (a, b), predicted‐experimental values (c, d), and perturbation plots (e, f) for protein yield and reducing sugar. *X*_1_: pH, *X*_2_: temperature (°C), *X*_3_: enzyme dosage (FBGU/g seed), *X*_4_: extraction time (h).

Figure 1 presents the response surface, predicted versus experimental, and perturbation plots describing the effects of enzymatic pretreatment variables on protein extraction yield and reducing sugar content. The three‐dimensional response surface plots (Figure 1a,b) illustrate the combined effects of extraction time and temperature on protein extraction yield and reducing sugar content. In both cases, a pronounced curvature of the response surfaces was observed, indicating the presence of significant quadratic effects and a well‐defined optimum region within the studied experimental range. Moderate increases in temperature and extraction time enhanced both responses, whereas further increases resulted in a gradual decline. The agreement between predicted and experimental values is presented in Figure 1c,d. The close clustering of data points around the parity line confirms the good predictive performance of the developed models for both protein extraction yield and reducing sugar content. Perturbation plots are effective tools for evaluating the relative influence of individual process variables on response parameters while keeping other factors constant (Koç et al. 2011). As shown in Figure 1e,f, protein extraction yield was more sensitive to changes in pH and enzyme dosage, whereas temperature and extraction time exhibited comparatively milder effects near the reference point. For reducing sugar content, enzyme dosage and temperature displayed steeper trends, highlighting their dominant role in polysaccharide hydrolysis during enzymatic pretreatment.

As a result of the simultaneous maximization of protein extraction yield and reducing sugar content, the optimal conditions for fennel seed enzymatic pretreatment were determined as pH 4.20, a temperature of 48.95°C, an enzyme dosage of 1.91 FBGU/g seed, and a treatment time of 2.60 h, with a desirability value of 1.00. Under these conditions, the predicted protein extraction yield and reducing sugar content were 92.33% and 104.17 mg glucose/g seed, respectively. Verification experiments conducted in triplicate yielded an experimental protein extraction yield of 92.22% ± 0.38%, which was not statistically different from the predicted value (*p* > 0.05), confirming the robustness of the optimization model.

Compared with the control sample obtained by classical alkaline extraction without enzymatic pretreatment, which exhibited a protein extraction yield of 61.22% ± 0.50%, enzymatic pretreatment markedly enhanced protein recovery from fennel seeds. This improvement can be attributed to the action of carbohydrase enzymes in Viscozyme L, which hydrolyze structural polysaccharides in the cell wall, thereby reducing matrix rigidity and facilitating protein solubilization. Similar enhancements in protein extraction efficiency following enzymatic pretreatment have been reported for oat bran, sesame meal, and rapeseed by‐products (Görgüç et al. 2019; Guan and Yao 2008; Rodrigues et al. 2014).

Following enzymatic pretreatment, classical alkaline extraction (pH 10.0, 1 h) resulted in a protein extraction yield of approximately 92%, indicating that enzymatic disruption of the fennel seed matrix substantially improved protein solubilization compared to the control sample obtained without enzymatic pretreatment (61.22% ± 0.50%). When UAE was applied after enzymatic pretreatment, a slightly higher protein yield (93.11% ± 0.82%) was achieved within only 10 min. This finding is particularly important, as it demonstrates that ultrasound can markedly shorten extraction time while maintaining comparable, or even marginally higher, protein recovery. Similar observations have been reported for different plant protein sources, where UAE enhanced protein yield at shorter processing times compared to conventional alkaline extraction, mainly due to improved cell disruption and mass transfer (Hildebrand et al. 2020; Sert et al. 2022). In addition, several studies have shown that UAE can be optimized to achieve high protein recovery with reduced processing time and energy demand, while also improving functional properties of the extracted proteins (Gong et al. 2025; Kibar et al. 2024; Sun et al. 2021). Similarly, Yeasmen and Orsat (2024) reported that pulsed UAE improved protein recovery from sugar maple leaves compared with conventional extraction, which they associated with enhanced cell disruption and mass transfer during sonication. Overall, the present results support the use of UAE as a practical and time‐efficient approach for fennel seed protein recovery.

The results demonstrate that enzymatic pretreatment is an effective strategy for improving protein extraction from fennel seeds and that the optimized conditions identified in this study are consistent with previously reported EAE systems. The observed trends are also in agreement with earlier findings on fennel and related plant materials, including the cress seed protein system reported by Turker and Isleroglu (2024).

### 3.2 Functional Properties of Fennel Seed Protein Isolate

**FIGURE 2:**
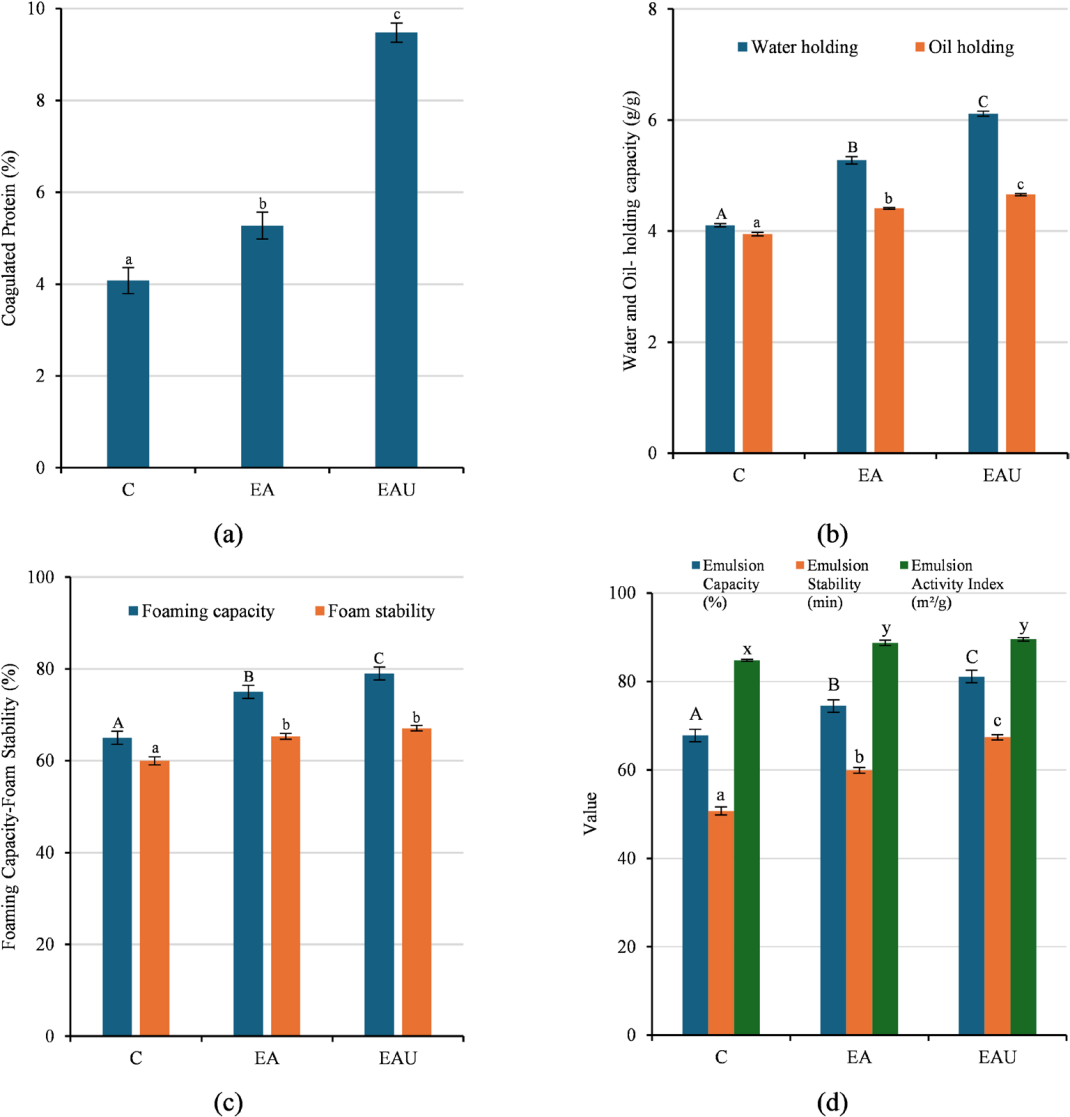
Functional properties of fennel seed protein isolates (a) coagulated protein, (b) water and oil holding capacities, (c) foaming properties, and (d) emulsion properties. Different letters above the bars indicate statistically significant differences among samples within each parameter (*p* < 0.05).

The functional properties of fennel seed protein isolates obtained by different extraction strategies are summarized in Figure 2. Overall, enzymatic pretreatment and UAE markedly improved the techno‐functional performance of the protein isolates compared to the control sample, indicating pronounced structure–function relationships.

Coagulated protein content reflects the thermal sensitivity of soluble proteins and is commonly used to assess heat‐induced aggregation behavior (Kramer and Kwee 1977). As shown in Figure 2a, the coagulated protein percentage increased from the control sample to EA and further to EAU. The highest value observed for the EAU sample suggests that ultrasound‐induced conformational rearrangements enhanced heat‐driven aggregation, likely due to partial unfolding and increased exposure of reactive sites. Similar ultrasound‐related increases in coagulation behavior have been reported for fenugreek and other plant protein isolates, where sonication promoted minor structural changes without severe denaturation (Akdeniz and Akalın 2019; Turker and Isleroglu 2024).

WHC and OHC are critical parameters governing the applicability of protein isolates in formulated foods. As illustrated in Figure 2b, both EA and EAU samples exhibited significantly higher water‐holding capacity than the control, with the maximum value recorded for EAU. A similar increase in WHC following UAE was also reported for sugar maple leaf protein by Yeasmen and Orsat (2024), who observed higher WHC values for ultrasound‐treated samples compared with conventionally extracted proteins (4.19 ± 0.95 vs. 3.52 ± 0.89 g/g). This enhancement can be attributed to increased molecular flexibility and the exposure of hydrophilic regions following enzymatic pretreatment and ultrasound, which facilitate water entrapment within the protein matrix. Comparable improvements in water‐holding capacity following enzyme–ultrasound treatments have been reported for fenugreek, cress seed, chickpea, and quinoa proteins (Perović et al. 2022; Turker et al. 2024; Yang et al. 2024).

A similar trend was observed for oil‐holding capacity (Figure 2b), where the EAU protein isolate exhibited the highest value. The enhanced oil‐binding ability is commonly associated with ultrasound‐induced exposure of hydrophobic amino acid residues, which increases affinity toward lipid phases (Gadalkar and Rathod 2020; Resendiz‐Vazquez et al. 2017). The concurrent improvement in both WHC and OHC highlights the dual amphiphilic character of the EAU protein isolate, which is highly desirable for complex food systems.

Foaming properties of the fennel seed protein isolates are presented in Figure 2c. Both FC and FS were significantly improved by enzymatic pretreatment and ultrasound application, with the highest values observed for the EAU sample (79.00% ± 1.41% for FC and 67.08% ± 0.59% for FS). These results were comparable with the previously reported foaming properties of different plant protein isolates. Feyzi et al. (2017) compared the functional properties of fenugreek ( *T. foenum‐graecum* ) protein isolates produced using different defatting solvents, and they reported the highest FC and FS of fenugreek seed protein isolate as ~31% and ~68%, respectively. Jambrak et al. (2009) investigated the physical properties of ultrasound‐treated soy protein isolate and reported the FC of untreated soy protein isolate as 110%, while ultrasound‐treated samples showed FC values ranging from 107% to 153%, depending on the ultrasound treatment conditions. Turker and Isleroglu (2024) reported that FC increased from 37% in the control cress seed protein isolate to 47% after enzyme–UAE, while FS increased from ~54% to ~62%. Compared with these reports, the EAU fennel seed protein isolate exhibited higher FC than fenugreek and cress seed protein isolates, while its FS was comparable to fenugreek protein isolate and slightly higher than enzyme–ultrasound‐treated cress seed protein isolate. Although its FC was lower than that reported for soy protein isolate, the simultaneous improvement in both FC and FS after the integrated enzyme–ultrasound treatment indicates enhanced air–water interfacial functionality. This improvement may be associated with increased molecular flexibility and surface hydrophobicity, which can promote protein adsorption at the air–liquid interface and support the formation of cohesive viscoelastic films (Mine 1995; Wang et al. 2020). Similar enhancements in foaming performance following UAE have been reported for fenugreek and chickpea protein isolates, supporting the trends observed in the present study (Turker and Isleroglu 2024; Wang et al. 2020). Therefore, the improved foaming behavior of the EAU sample supports the potential applicability of fennel seed protein isolate in aerated food formulations.

The emulsification properties of the protein isolates, including ES, EC, and EAI, are shown in Figure 2d. Among all samples, the EAU protein isolate consistently exhibited superior emulsification performance. For comparison, Jiang et al. (2009) reported that soy protein isolate had an EAI of 70.1 m^2^/g and an ES index of 41.3 min under control conditions, which increased to 91.5 m^2^/g and 55.8 min after alkaline pH‐shifting, respectively. Turker and Isleroglu (2024) reported that enzyme–UAE improved the emulsifying properties of cress seed protein isolate, increasing ES from 19.64 to 20.23 min, emulsion activity from 71.53 to 72.09 m^2^/g, and EC from 39.44% to 49.44%. Feyzi et al. (2018) reported that optimized grass pea protein isolate produced based on extraction yield showed higher emulsifying capacity (87.50%) than the EAU fennel seed protein isolate, while its ES was 28.65%. Feyzi et al. (2017) also compared fenugreek protein isolates produced using different defatting solvents and reported EAI, EC, and ES index values up to 93.28 m^2^/g, 24.42%, and 22.23 min, respectively. These comparisons indicate that the emulsifying performance of fennel seed protein isolate was within the range reported for several plant protein isolates, although the relative performance varied depending on the emulsion parameter considered. In particular, the EAU sample showed improved ES and capacity compared with the control, supporting the positive effect of the integrated treatment on emulsifying functionality.

The improvement observed in the EAU sample can be attributed to the combined effects of enzymatic pretreatment and UAE. Enzymatic pretreatment may facilitate protein release by weakening the seed matrix, while ultrasound treatment can induce moderate conformational modifications, increase molecular flexibility, and promote the exposure of surface‐active groups. These changes may enhance protein adsorption at the oil–water interface and strengthen protein–protein interactions around oil droplets, thereby improving ES and EC. Similar synergistic effects of enzymatic pretreatment and ultrasound on the emulsifying properties of plant proteins have also been reported in previous studies (Perović et al. 2020; Turker and Isleroglu 2024; Yang et al. 2024).

The results demonstrate that the combination of enzymatic pretreatment and UAE not only enhances protein recovery but also significantly improves the functional performance of fennel seed protein isolates. Compared to the previously reported cress seed protein systems, fennel seed proteins exhibited comparable or superior water/oil binding, foaming, and emulsification behaviors, highlighting their strong potential as multifunctional ingredients for food formulations.

**FIGURE 3:**
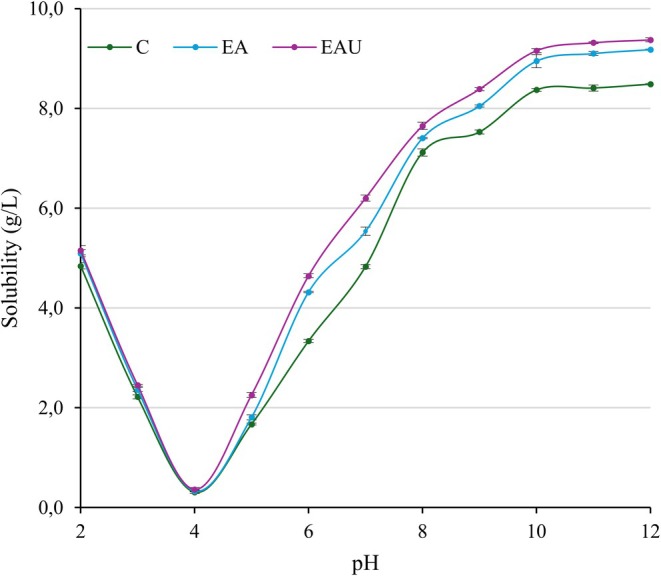
Solubility graph for fennel seed protein isolates.

The pH‐dependent solubility profiles of fennel seed protein isolates obtained by different extraction methods are presented in Figure 3. All samples exhibited a typical U‐shaped solubility pattern, with minimum solubility observed around pH 4.0 and a gradual increase toward both acidic and alkaline regions. The lowest solubility values were recorded at pH 4.0 for the control (0.31 g/L), EA (0.33 g/L), and EAU (0.36 g/L) samples, whereas maximum solubility was achieved at pH 12.0, reaching 8.49, 9.18, and 9.37 g/L for the respective samples. For comparison, Gao et al. (2022) reported a solubility of 7.20 g/L for untreated commercial pea protein isolate. In addition, Zhang et al. (2024) reported that the protein solubility of commercial soy protein isolates ranged from 36.99% to 81.87% when 0.2 g of SPI was dispersed in 20 mL of phosphate buffer. Based on this dispersion concentration, the highest solubility value corresponds to approximately 8.19 g/L. Feyzi et al. (2018) reported 9.2 g/L solubility for freeze‐dried grass pea protein isolate, and Turker and Isleroglu (2024) reported solubility of cress seed protein isolate as 7.90 g/L. These comparisons suggest that the maximum solubility of the EAU fennel seed protein isolate (9.37 g/L) was within a comparable range and slightly higher than several reported values for commonly studied plant protein isolates. This indicates that fennel seed protein isolate exhibited favorable solubility characteristics, particularly under alkaline conditions, supporting its potential use in food formulations requiring good protein dispersibility. The minimum solubility around pH 4.0 suggests that the isoelectric point of fennel seed proteins is close to this value. At the isoelectric point, proteins carry a net zero charge, resulting in reduced electrostatic repulsion and enhanced protein–protein interactions, which promote aggregation and precipitation. In contrast, under acidic or alkaline conditions, proteins acquire positive or negative charges, increasing electrostatic repulsion and hydration, thereby enhancing solubility (Aluko 2024; Li et al. 2020; Martínez‐Flores et al. 2006). Similar solubility behavior has been reported for soybean, rapeseed, and other oilseed protein isolates, where minimum solubility occurs near pH 4.0–5.0 and increases markedly at alkaline pH values (Ge et al. 2021; Gerzhova et al. 2015; Lam et al. 2018).

Among the samples, the EAU protein isolate consistently exhibited higher solubility across the entire pH range, with the most pronounced difference observed under alkaline conditions (Figure 3). The enhanced solubility of EAU samples can be attributed to ultrasound‐induced structural modifications, including particle size reduction, partial unfolding of protein molecules, and the exposure of hydrophilic groups, which collectively improve protein–water interactions. Comparable increases in protein solubility following ultrasound treatment have been reported for plant and dairy proteins, including pea, whey, and moringa seed proteins (Fatima et al. 2023; Jambrak et al. 2009; Jiang et al. 2017). Overall, these findings indicate that the combination of enzymatic pretreatment and ultrasound is effective in improving the solubility of fennel seed proteins, particularly under alkaline conditions, which is desirable for many food applications.

### 3.3 Molecular, Structural, and Thermal Properties of Protein Isolates

#### 3.3.1 Molecular Weight Distribution of Fennel Seed Protein Isolates

**FIGURE 4:**
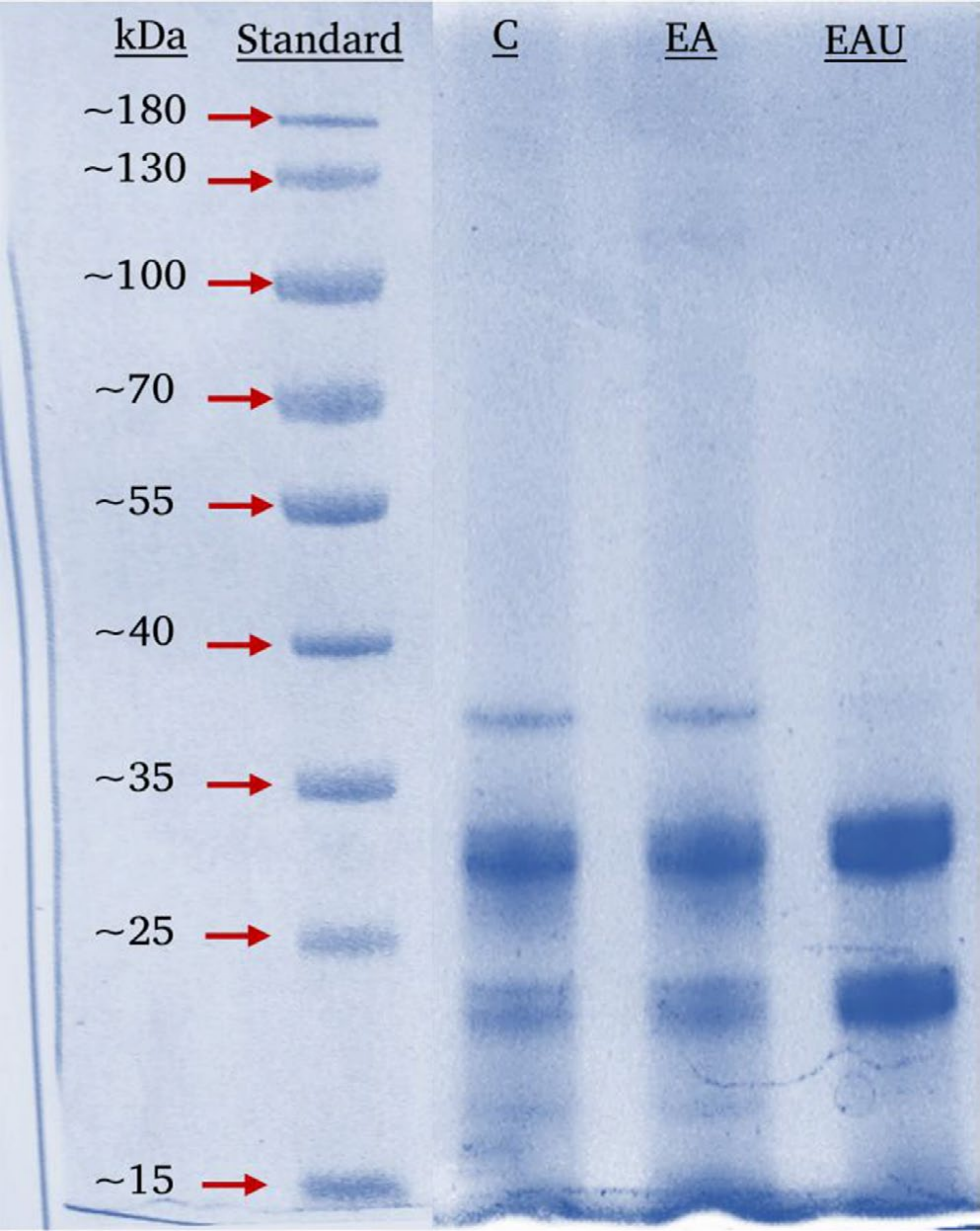
SDS‐PAGE gel image of the fennel seed proteins. For PageRuler prestained protein ladder (10–180 kDa), only visible marker bands are indicated on the gel image.

The molecular weight distribution of fennel seed protein isolates was evaluated using SDS‐PAGE (Laemmli 1970) (Figure 4). Similar electrophoretic patterns were observed for all samples, indicating that the applied extraction strategies did not markedly alter the major protein fractions.

Fennel seed protein isolates exhibited molecular weight bands ranging approximately from 20 to 120 kDa, with distinct bands detected around 20, 22, 23, 24, 32, and 37 kDa. Bands within the 22–70 kDa range are typically associated with globulin‐type storage proteins, particularly legumins and vicilins, which constitute the dominant protein fractions in many oilseed matrices (Felix et al. 2019; Mulla and Ahmed 2019). Proteins in this molecular weight range are known to contribute to key technofunctional properties such as emulsification, foaming, and gelation, and may also serve as precursors of bioactive peptides with nutritional relevance (Karami and Akbari‐adergani 2019; Sânchez‐Vioque et al. 1999). A comparable molecular weight distribution was previously reported for cress seed protein isolates produced using similar extraction strategies, where the major protein bands were also concentrated within the globulin‐associated region and were largely unaffected by enzymatic pretreatment and ultrasound application (Turker and Isleroglu 2024). The consistency observed between fennel and cress seed protein isolates suggests that enzymatic and ultrasound‐assisted processes mainly influence protein conformation and functionality rather than causing extensive protein fragmentation.

#### 3.3.2 Structural Properties of Fennel Seed Protein Isolates

**FIGURE 5:**
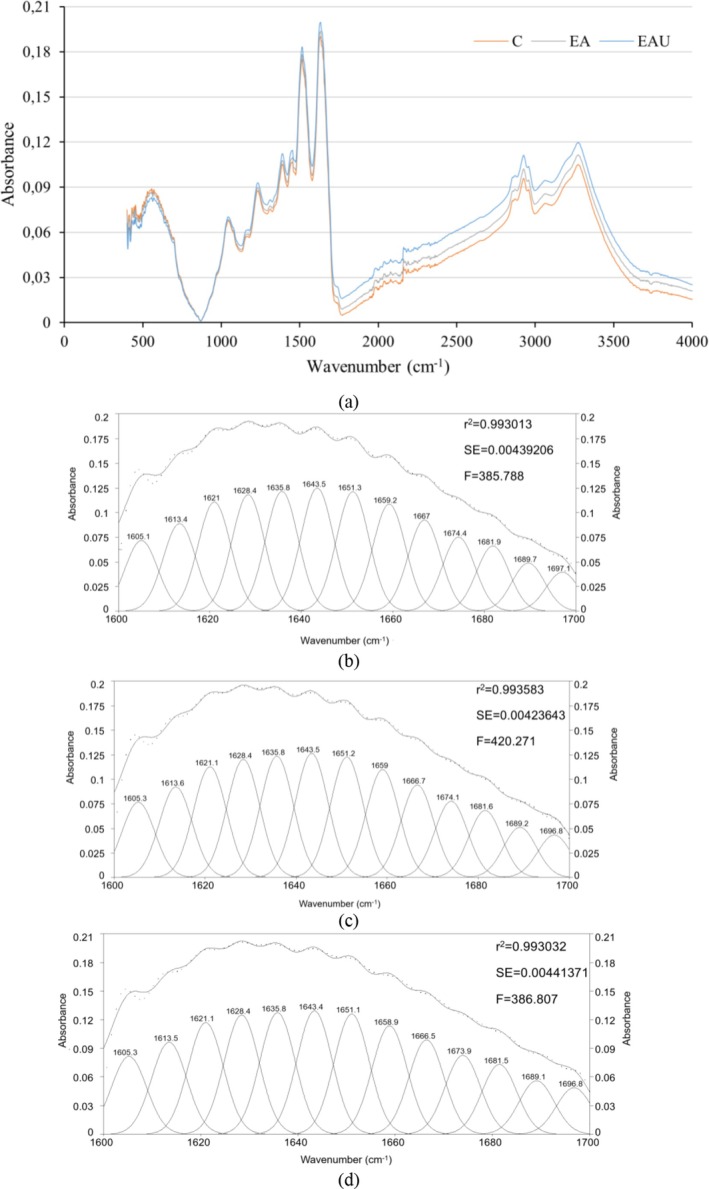
Structural properties (a) FT‐IR spectra and the distribution of peaks in the Amide I region of the fennel seed protein isolates (b) control, (c) EA, and (d) EAU.

FT‐IR analysis revealed that all fennel seed protein isolates exhibited comparable spectral patterns, suggesting that the applied extraction strategies did not induce major alterations in the overall protein backbone (Figure 5a). The presence of a well‐defined Amide I band (1600–1700 cm^−1^) in all samples confirmed the suitability of this region for secondary structure evaluation (Kong and Yu 2007). In addition, Amide II (1480–1585 cm^−1^) and Amide III (1200–1400 cm^−1^) bands were detected, indicating preserved peptide structures and protein–carbohydrate interactions (Barth and Zscherp 2002; Withana‐Gamage et al. 2011).

Deconvolution of the Amide I region demonstrated that β‐sheet structures were predominant in all fennel seed protein isolates (Figure 5b–d). The β‐sheet content increased from 39.01% in the control sample to 39.41% after enzymatic pretreatment and further to 43.34% following the combined enzyme–ultrasound treatment. This increase was accompanied by a gradual reduction in side‐chain contributions (from 5.43% to 2.15%) and a slight decrease in β‐turn structures (from 25.76% to 22.33%), while α‐helix content showed only minor variations (18.95%–20.28%). Random coil fractions remained relatively stable across all samples (10.85%–11.90%). These results indicate that ultrasound application induced moderate conformational rearrangements rather than extensive unfolding, leading to a more ordered secondary structure dominated by β‐sheets.

#### 3.3.3 Thermal Properties of Fennel Seed Protein Isolates

**FIGURE 6:**
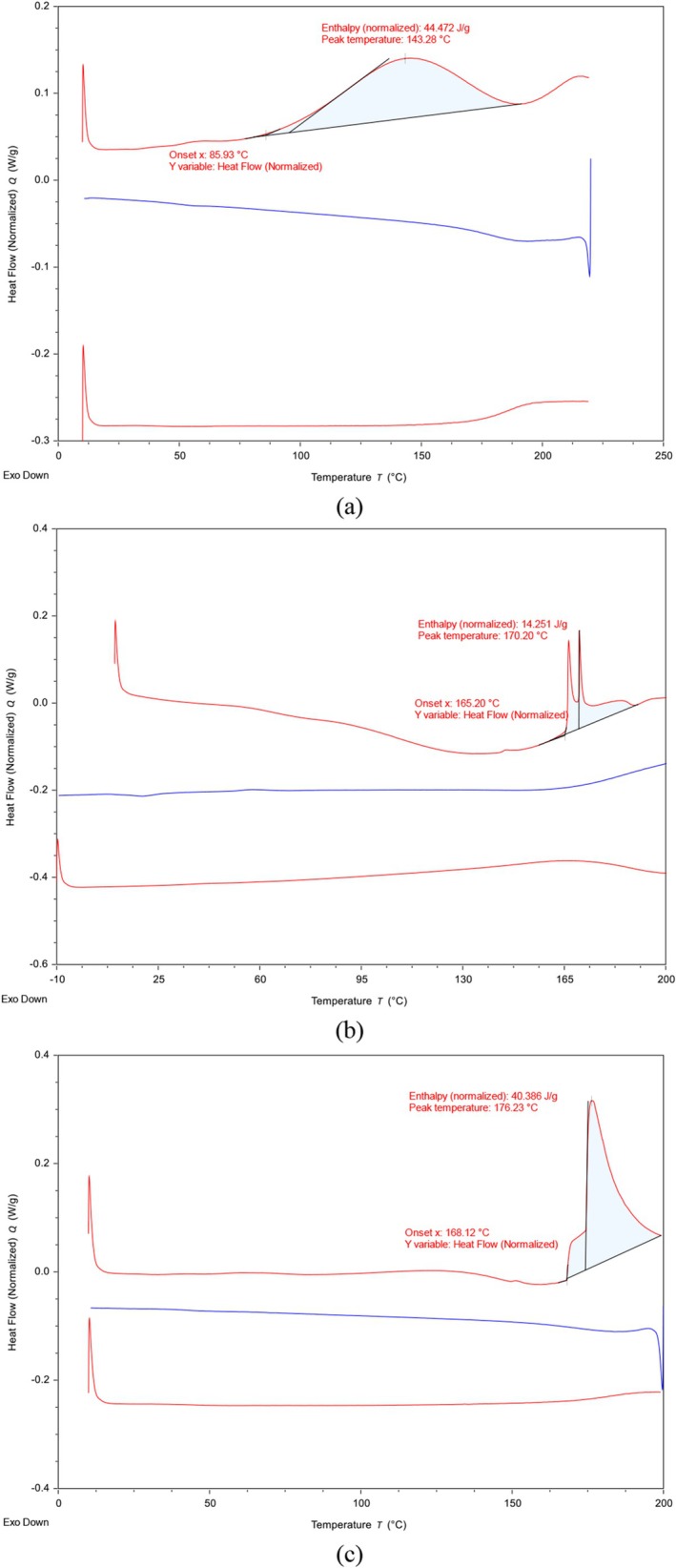
DSC thermograms for different fennel seed protein isolates (a) control, (b) EA, and (c) EAU.

The thermal behavior of fennel seed protein isolates obtained by different extraction strategies was evaluated by DSC, and the corresponding thermograms are presented in Figure 6. All samples exhibited clear endothermic transitions, indicating that energy input was required for protein denaturation.

The denaturation temperature (*T*_d_) of the control protein isolate was recorded as 143.28°C, whereas higher *T*_d_ values were recorded for the EA and EAU samples, reaching 170.20°C and 176.23°C, respectively. This progressive increase in denaturation temperature suggests that enzymatic pretreatment, particularly when combined with ultrasound, enhanced the thermal stability of fennel seed proteins. Similar shifts toward higher denaturation temperatures have been reported for plant protein isolates subjected to enzymatic and ultrasonic modifications, which were attributed to changes in protein–protein interactions and molecular organization (Deng et al. 2019; Olivos‐Lugo et al. 2010).

The denaturation enthalpy (Δ*H*_d_) values were determined as 44.72 J/g for the control sample, 14.25 J/g for EA, and 40.39 J/g for EAU protein isolates. The relatively lower Δ*H*_d_ value observed for the EA sample may indicate partial unfolding or disruption of ordered structures during enzymatic pretreatment, whereas the higher enthalpy requirement of the EAU sample suggests the formation of a more compact or reorganized protein structure requiring greater energy for denaturation. Comparable trends have been reported for ultrasound‐treated plant proteins, where sonication was shown to induce molecular rearrangements and modify the balance between hydrophobic interactions and hydrogen bonding (Rashid and Gülseren 2024; Shevkani et al. 2015).

The enhanced thermal stability observed for the EAU protein isolate is consistent with the FT‐IR results, which indicated a higher proportion of β‐sheet structures in this sample. β‐sheet‐rich protein conformations have been associated with increased resistance to thermal unfolding due to stronger intermolecular interactions (Wang et al. 2020). Similar relationships between secondary structure composition and thermal behavior have been reported for basil, flaxseed, and fenugreek protein isolates (Feyzi et al. 2015; Rashid and Gülseren 2024; Tang et al. 2006). Overall, the DSC results demonstrate that the combination of enzymatic pretreatment and ultrasound not only modifies the structural organization of fennel seed proteins but also improves their thermal stability, which is a desirable attribute for potential applications in thermally processed food systems.

### 3.4 Amino Acid Composition of Fennel Seed Protein Isolates

The amino acid composition of fennel seed protein isolate (EAU sample) is presented in Table 3 and compared with hen's egg and soy protein isolate as reference proteins. The total essential amino acid content of fennel seed protein isolate was 40.86 g/100 g protein, indicating a nutritionally rich profile among plant‐derived proteins. Leucine (8.26 g/100 g), lysine (9.10 g/100 g), valine (5.67 g/100 g), and phenylalanine (5.63 g/100 g) were the predominant essential amino acids, whereas threonine and methionine were present at relatively lower levels. A similar imbalance of sulfur‐containing amino acids has been widely reported for plant protein isolates and is considered a common limitation compared to animal proteins (Galili and Amir 2013; Gorissen et al. 2018).

**TABLE 3:** Amino acid composition of fennel seed protein isolate obtained by enzymatic pretreatment followed by ultrasound‐assisted extraction (EAU).

	Amino acid	Fennel seed protein isolate	Hen's egg	Soy protein isolate	Amino acid score
Essential amino acids (g amino acid/100 g protein isolate)	Histidine	2.42	2.40	2.53	1.01
	Threonine	2.77	5.10	3.86	0.54
	Valine	5.67	7.50	4.80	0.76
	Methionine	1.74	3.20	1.26	0.54
	Phenylalanine	5.63	5.10	4.94	1.10
	Isoleucine	5.28	5.60	4.54	0.94
	Leucine	8.26	8.30	7.78	1.00
	Lysine	9.10	6.20	6.38	1.47
	Total	40.86	46.40	36.09	—
Nonessential amino acids (g amino acid/100 g protein isolate)	Tyrosine	2.76	4.00	3.14	0.93
	Aspartic acid	1.79	10.70	11.70	0.21
	Glutamic acid	4.67	12.00	18.70	0.39
	Serine	2.39	7.90	5.49	0.33
	Glycine	3.29	3.00	4.18	1.33
	Arginine	4.72	6.10	7.23	1.02
	Alanine	4.05	5.40	4.26	1.34
	Proline	3.89	3.80	5.49	0.93
	Cysteine	—	1.80	1.33	—
	Tryptophan	1.10	—	1.28	—
	Total	34.45	54.70	62.80	—

Amino acid scores were calculated using hen's egg protein as the reference. Scores close to or above unity were obtained for histidine, phenylalanine, leucine, and lysine, with lysine showing a notably high score (1.47), highlighting the nutritional complementarity of fennel seed protein with cereal‐based foods that are typically deficient in lysine. Comparable trends have been reported for cress seed protein isolate, where relatively high lysine and branched‐chain amino acid contents contributed to improved nutritional quality when compared with other plant protein sources (Turker and Isleroglu 2024).

When compared with soy protein isolate, fennel seed protein exhibited similar essential amino acid levels for leucine, isoleucine, and phenylalanine, while showing higher lysine content. The nonessential amino acid fraction was dominated by glutamic acid, arginine, alanine, and glycine, which are known to contribute to protein functionality as well as nutritional value (Table 3). Amino acid composition suggests that fennel seed protein isolate represents a valuable plant‐based protein source with balanced essential amino acid content and potential for use in functional foods and protein‐enriched formulations.

## 4 Conclusion

This study demonstrates that fennel seeds represent a promising and underexplored plant source to produce functional protein isolates. The application of enzyme‐assisted pretreatment significantly enhanced protein extraction efficiency by disrupting the polysaccharide‐rich seed matrix, while subsequent UAE enabled comparable or slightly higher protein yields within a markedly reduced processing time compared to classical alkaline extraction. The optimized enzymatic conditions resulted in high extraction efficiency, and the integration of ultrasound further improved process sustainability without inducing severe structural degradation. Functional characterization revealed that protein isolates obtained via the combined enzymatic and ultrasound‐assisted approach exhibited superior solubility, emulsifying, foaming, and water/oil‐holding capacities relative to control samples. Structural analyses confirmed that these functional improvements were associated with minor conformational rearrangements rather than extensive protein denaturation. The predominance of β‐sheet structures and the elevated denaturation temperatures indicated good thermal stability of the fennel seed protein isolates. In addition, amino acid profiling highlighted a balanced essential amino acid composition, supporting the nutritional value of the extracted proteins. The findings indicate that the integration of enzymatic pretreatment and UAE is an effective, time‐efficient, and environmentally compatible strategy for producing high‐quality fennel seed protein isolates. These protein isolates show strong potential for incorporation into a wide range of food formulations, particularly in plant‐based and functional food systems. Nevertheless, further studies are needed to evaluate their performance in different food matrices and processing conditions.

## Author Contributions

**Hilal Isleroglu:** conceptualization, supervision, writing – review and editing, methodology. **Izzet Turker:** data curation, investigation, formal analysis, writing – original draft.

## Funding

This study was financially supported by Tokat Gaziosmanpasa University Scientific Research Projects Committee (Project No: 2022/22).

## Ethics Statement

The authors have nothing to report.

## Consent

The authors have nothing to report.

## Conflicts of Interest

The authors declare no conflicts of interest.

## Data Availability

The data that support the findings of this study are available from the corresponding author upon reasonable request.
